# Impacts of Climate-Change-Driven Sea Level Rise on Intertidal Rocky Reef Habitats Will Be Variable and Site Specific

**DOI:** 10.1371/journal.pone.0086130

**Published:** 2014-01-22

**Authors:** Jaqueline Thorner, Lalit Kumar, Stephen D. A. Smith

**Affiliations:** 1 University of New England (UNE), School of Rural and Environmental Sciences, Armidale, New South Wales, Australia; 2 National Marine Science Centre, Southern Cross University (SCU), Coffs Harbour, New South Wales, Australia; University of Vigo, Spain

## Abstract

Intertidal rocky reefs are complex and rich ecosystems that are vulnerable to even the smallest fluctuations in sea level. We modelled habitat loss associated with sea level rise for intertidal rocky reefs using GIS, high-resolution digital imagery, and LIDAR technology at fine-scale resolution (0.1 m per pixel). We used projected sea levels of +0.3 m, +0.5 m and +1.0 m above current Mean Low Tide Level (0.4 m). Habitat loss and changes were analysed for each scenario for five headlands in the Solitary Islands Marine Park (SIMP), Australia. The results indicate that changes to habitat extent will be variable across different shores and will not necessarily result in net loss of area for some habitats. In addition, habitat modification will not follow a regular pattern over the projected sea levels. Two of the headlands included in the study currently have the maximum level of protection within the SIMP. However, these headlands are likely to lose much of the habitat known to support biodiverse assemblages and may not continue to be suitable sanctuaries into the future. The fine-scale approach taken in this study thus provides a protocol not only for modelling habitat modification but also for future proofing conservation measures under a scenario of changing sea levels.

## Introduction

There is strong consensus that sea levels will rise by as much as 1 m by 2100 in response to a warming climate [1]. While sea levels have varied by >120 m during the glacial/interglacial cycles, there was little change over the past several thousand years [2]. However, this condition of relative stasis changed during the 19th and early 20th centuries when the first indications of a rising sea level became evident [2], [3]. Recent sea level rise (SLR) is a result of both ocean thermal expansion and the melting of glaciers and ice caps, and accelerated rates of SLR are likely to be largely attributable to the liberation of water comprising polar ice sheets [2], [4]–[6]. According to Church *et al*. [7] an upper limit of 0.88 m in mean sea level is expected over the next hundred years. However, recent data acquired from satellite-altimeter and tide-gauge sources suggest sea levels are rising at over 3 mm/yr [8] with an acceleration rate of 0.013 mm/yr [9]. Current rates of SLR are at the upper limit of the projections of the Third Assessment Report of the Intergovernmental Panel on Climate Change (IPCC) [7], and there is strong concern that the contribution of shrinking ice sheets will push these even higher, especially if greenhouse gas emissions continue to increase [2]. Most of the climate models indicate that the increase in temperature over Greenland is one to three times higher than the global average. Ice sheet models project that a local warming of more than 3°C, if sustained over a longer term, would lead to the complete melting of the Greenland ice sheet, adding around 7 m to the sea level [10]. Also, the West Antarctic Ice Sheet could potentially contribute about 6 m to the sea level. The entire Antarctic ice sheet holds enough water to raise global sea levels by 62 m [5]. As current predictive models have a high level of uncertainty, there is concern that the estimates of mean SLR by 2100 could be considerably underestimated [7], [11]. Future SLR is not expected to be globally uniform due to ocean circulation, wind pressure patterns and geological uplift [7], [11]–[14]. Effects operating over local or regional scales, such as storm surges and spring tides, will add further variability [11].

While the magnitude of SLR is clearly difficult to predict with certainty, there is little doubt that intertidal habitats are likely to be the first to experience sea level rise-related effects [15]. Rocky shores occupy 30% of the world's coastline [16] and are complex and rich ecosystems supporting high biodiversity [17]. As one of the most accessible, diverse marine habitats, rocky shores are important features for education, recreation and harvesting [17]–[19]. When compared to other intertidal habitats such as beaches, mangroves and estuaries, and due to their geological nature, erosion processes are relatively slow on intertidal rocky reefs obviating the creation of new habitat over short periods of time: this exposes the biota to the very real threat of habitat loss with consequent reduction in biodiversity due to “coastal squeeze” [19]. Indeed, habitat loss and change is potentially one of the greatest threats to biodiversity conservation in a changing world [20], [21], and is a key challenge for the management of biodiversity through static systems such as Marine Protected Areas (MPA) [22], [23].

Predicting the magnitude of changes to intertidal rocky reefs, however, relies not only on accurate predictions of SLR, but also on the development of technologies to map and quantify habitats and environmental processes at appropriate spatial scales, in an efficient and cost-effective way [24]. Tools such as remote sensing and modelling, are becoming increasingly available [8], [25]–[27] and are already being applied to the assessment of risk associated with SLR [28]. To date, LIDAR (Light Detection and Ranging) has the highest horizontal resolution and vertical accuracy [24], [29] and is the only technology suitable for analyzing sea-level changes in the range predicted for the next 100 years [30], [31].

Following the development and assessment of a combined approach using LIDAR, high-resolution digital imagery and GIS [31], this study evaluated the fine-scale consequences of different projections of sea level on the range of intertidal habitats for five headlands in the Solitary Islands Marine Park (SIMP), northern NSW, Australia. Specifically, we evaluated seven categories of rocky shore habitat (lower shallow pools, upper shallow pools, deep pools, upper boulder fields, lower boulder fields, upper platform and lower platform) and the current area of each habitat type was compared to future modelled scenarios to determine the likely changes to these intertidal rocky ecosystems and the possible consequences for biodiversity conservation.

### Study Area

**Figure 1:**
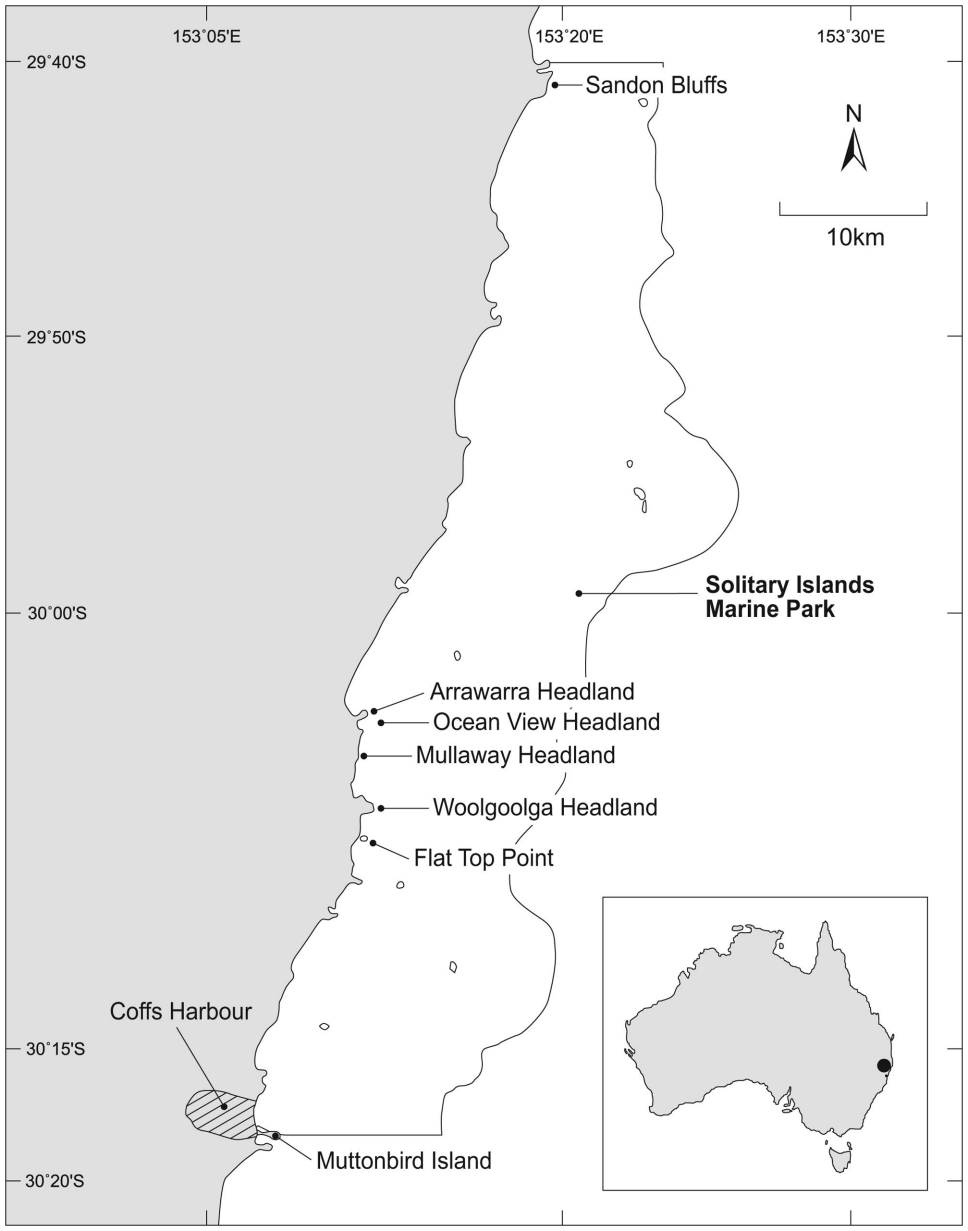
Solitary Island Marine Park map showing the five headlands analyzed in this study. Arrawarra Headland; Ocean View Headland; Mullaway Headland; Woolgoolga Headland and Flat Top Point.

The SIMP is located on the mid-north-coast of NSW, Australia. The five headlands chosen for this research were: Arrawarra Headland (153°12′07′′E-30°3′33′′S); Oceanview Headland (153°12′14′′E-30°04′01′S); Mullaway Headland (153°12′15′′E-30°4′33′′S); Woolgoolga Headland (153°12′17′′E-30°06′30′′S); and Flat Top Point (153°12′26′′E-30°07′48′′S) (Figure 1). The region has a 2 m, semi-diurnal tidal cycle, a Mean Sea Level of 0.9 m, a Mean Low Tide Level of 0.4 m, and a Mean High Tide Level of 1.4 m [32]. Each of the five headlands is composed of metamorphic greywacke deposits from the late Carboniferous, approximately 280–350 million years old [33]. The headland landscape comprises cliffs, bedrock and scree (boulder fields) with variable wave-driven erosional patterns and areas of sand accumulation. The region is renowned for the overlap of tropical and temperate currents [34], facilitating a high biodiversity due to the presence of tropical, temperate and endemic species [35]–[38]. There have been few published studies on the biodiversity and ecology of rocky shore habitats within the region, but those that have been conducted, indicate a high diversity, and distribution patterns that are largely driven by habitat type and exposure to wave energy [36]. The total intertidal rocky shore mapped area for each headland was: Arrawarra Headland (19.129 m^2^); Oceanview Headland (10.490 m^2^); Mullaway Headland (16.134 m^2^); Woolgoolga Headland (41.173 m^2^); and Flat Top Point (13.458 m^2^).

## Methods

### Habitat Mapping

Three-dimensional digital habitat maps were compiled for each of the five intertidal rocky reefs. The maps were obtained by coupling topographic LIDAR data (wavelength  = 1550 µ; Leica ASL60 system) and high-resolution imagery in ArcGIS software [31], [39]. The data resolution per pixel was 0.1 m (z), 0.3 m (x,y) for the LIDAR, and 0.1 m for the digital imagery. Intertidal rocky reef habitats were classified into seven types of habitats (Table 1) which recognized broad habitat type [36] and tidal height. Mean Sea Level (0.9 m) was used to differentiate between upper and lower shore levels. Ground-truthing of the remote-sensing data was performed along the headlands at very low tides (<0.4 m) on days with low swell (<1 m).

**Table 1:** Habitat classification based on the tidal exposure and substrate type (Source: [31]).

Habitat Classification	Habitat Description (Mean Sea Level (MSL) = 0.9-m)
Upper Boulder Field	≥2 boulders (>0.25-m) per m^2^ and located above MSL
Lower Boulder Field	≥2 boulders (>0.25-m) per m^2^ and located below MSL
Upper Shallow Pool	Rock pools no deeper than 0.5-m, with bottom composed
	of sand, cobble, boulder or a mix of these, and located
	above MSL
Lower Shallow Pool	Rock pools no deeper than 0.5-m, with bottom composed
	of sand, cobble, boulder or a mix of these, usually with
	high algal cover and located below MSL
Deep Pool	Rock pools deeper than 0.5-m, with bottom composed of
	sand, cobble, boulder or a mix of these (this habitat type
	only occurred at the seaward edge of the lower shore at
	the five study locations).
Upper Platform	Emerged bedrock located above MSL
Lower Platform	Emerged bedrock located below MSL

### Sea Level Rise Modelling

Flooding maps were created as a layer over the five original habitat maps using ArcMap [39]. SLR projections were based on the predictions from the 2007 IPCC report [30]. The basic tide level for the analysis was Mean Low Tide Level (MLTL) (0.4 m) with flooding maps created for SLR of 0.3 m, 0.5 m and 1.0 m above current levels [32]. Changes in habitat area under each SLR scenario were calculated using ArcMap [39] for each headland, and data were summarized both as habitat maps and as relative percentage change for each habitat type at each headland. It should be noted that we modelled only eustatic change: storm surges or extreme events were not taken into consideration. Predictions were made under the assumption that tidal pattern will not change over the next 100 years (1 m SLR). The ratio of upper and lower shore area was calculated in order to evaluate the change in relative distribution of habitat under the different sea levels for the five headlands. This was done because lower shores generally have higher species richness and changes in the availability of habitat on upper and lower shores are therefore likely to influence patterns of biodiversity [40]. The quantity, mean size (±SE) of pools in the upper and lower shallow pool habitats were calculated in order to evaluate the change in habitat quality over the prediction period.

## Results

### Change in the intertidal rocky reef landscape

**Figure 2:**
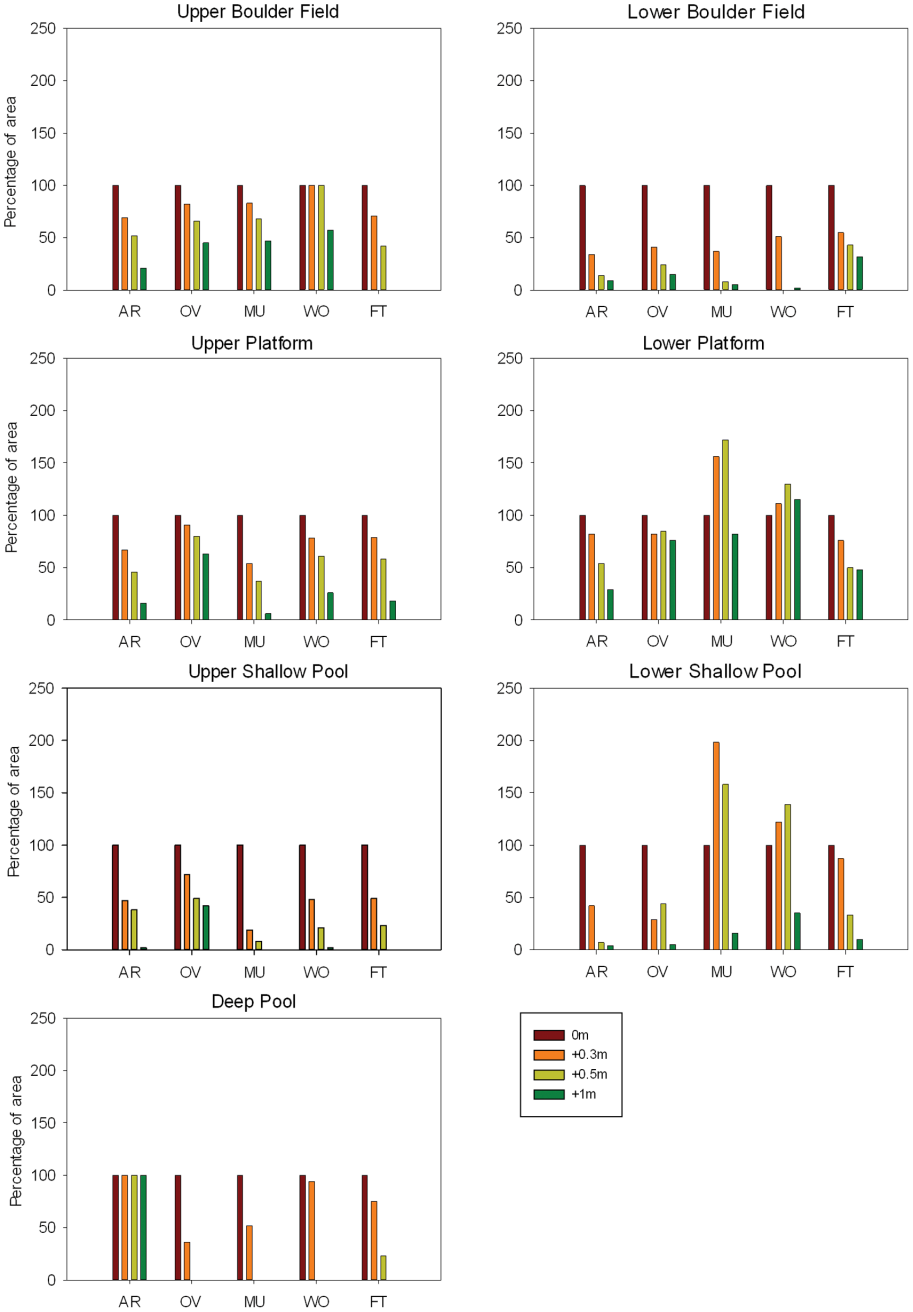
Habitats area change represented as percentage relative to current sea level at the five headlands. The four projected sea levels were 0+0.3 m,+0.5 m and 1 m and the five headlands AR-Arrawarra Headland; OV-Ocean View Headland; MU-Mullaway Headland; WO-Woolgoolga Headland; FT-Flat Top Point.

The results from the SLR projections show a dramatic change in the rocky shore landscape under a 1-m SLR, with an overall decline in habitat area for all habitat types with rare exceptions (Figure 2). Deep pools will be by far the most affected habitat type, disappearing from all headlands except Arrawarra under 1-m SLR. Upper shallow pools will be the second most affected habitat followed by lower boulder fields. Lower platform and lower shallow pool habitats will have an irregular pattern of change, with alternating increases and decreases in habitat area over the projection period. However, lower shallow pools will suffer a large loss under 1-m SLR, whereas lower platform habitat will suffer the least overall loss. Upper boulder field will be the second least affected habitat type followed by upper platform habitat. The change in the relative distribution of upper and lower habitats will also be irregular over the projected sea levels (Table 2). Arrawarra Headland and Ocean View Headland will show an initial increase in upper shore area with a subsequent decline at 0.3 m and 0.5 m SLR, nevertheless the final model predicts a larger upper shore area under 1 m SLR than at present. All the remaining headlands show a steady increase in the area of lower shore relative to the upper shore as sea level rises.

**Table 2:** Relative upper/lower shore area modification for the four projected sea levels at the five headlands.

Headland	0m	+0.3m	+0.5m	+1m
Arrawarra Headland	0.5	0.7	0.9	0.6
Ocean View Headland	2.5	3.7	3.5	3.4
Mullaway Headland	1.3	0.8	0.6	0.4
Woolgoolga Headland	2.7	2	1.4	0.7
Flat Top Point	1.4	1.1	1.3	0.4

### Upper Shallow Pool Habitat

Our modelling indicated that there will be an overall decline in the total area of upper shallow pool habitat at each of the five headlands under 1-m SLR. A reduction by >50% of habitat area will occur under 0.3-m SLR at all headlands except Ocean View Headland. At Mullaway Headland and Flat Top Point, the upper shallow pool habitat will completely disappear under 1-m SLR, whilst at Arrawarra Headland and Woolgoolga Headland, only 2% of the habitat will remain. Habitat loss will be lowest at Ocean View Headland which will maintain nearly half of its upper shallow pool habitat area under 1-m SLR. However, the habitat quality tends to change with SLR, with a decrease in the number of pools for all headlands. The mean size of pools (m^2^) will be reduced at Arrawarra Headland, Mullaway Headland and Flat Top Point whereas Ocean View Headland and Woolgoolga Headland will show a variable change in pool size as sea level rises (Table 3).

**Table 3:** Changes in the number of pools and their mean size (± SE) for the four projected sea levels at the five headlands (m^2^).

Headland	Sea Level	Upper Shallow Pool		Lower Shallow Pool	
		n	Mean ± SE	n	Mean ± SE
Arrawarra Headland	0m	32	5.57±0.79	60	25.82±5.24
	+0.3m	21	3.96±0.66	32	20.14±5.73
	+0.5m	18	3.82±0.7	14	7.81±1.36
	+1m	1	4.34±4.34	17	3.79±0.74
Ocean View Headland	0m	17	10.21±3.34	19	10.73±3.45
	+0.3m	13	9.59±3.88	6	9.98±4.5
	+0.5m	9	9.31±5.36	8	11.20±3.71
	+1m	6	12.28±7.75	3	3.38±0.8
Mullaway Headland	0m	48	16.21±5.11	44	8.86±1.09
	+0.3m	13	11.63±5.73	46	16.77±5.12
	+0.5m	11	5.85±1.29	36	17.15±6.36
	+1m	−	−	11	5.85±1.29
Woolgoolga Headland	0m	106	12.42±2.21	61	11.75±1.79
	+0.3m	57	11.18±2.37	69	12.68±2.88
	+0.5m	29	9.66±2.54	72	13.81±3.07
	+1m	2	11.9±5.41	27	9.5±2.69
Flat Top Point	0m	24	9.85±2.09	20	27.73±5.38
	+0.3m	19	6.12±1.03	19	25.32±5.12
	+0.5m	10	5.44±1.13	15	12.13±3.14
	+1m	−	−	10	5.44±1.13

### Lower Shallow Pool Habitat

Lower shallow pool habitat shows an irregular pattern of modification under 1-m SLR. At all headlands, with the exception of Woolgoolga Headland, habitat extent will be reduced by >80% under 1-m SLR. At Mullaway and Woolgoolga headlands, SLR will initially lead to an increase in lower shallow pool habitat (under 0.5-m SLR), with a subsequent decline. Ocean View Headland shows a dramatic reduction, to nearly 29% of the current extent, under 0.3-m SLR, followed by an increase to 44% under 0.5-m SLR, and a further decline to 5% under 1-m SLR. Flat Top Point shows the greatest reduction of habitat (50%) between 0.3-m and 0.5-m SLR. The number of pools will be reduced at all headlands; however, the mean pool size will tend to a steady decrease only in Arrawarra Headland, Ocean View Headland and Flat Top Point. Mullaway Headland and Woolgoolga Headland will initially show an increase in mean pool size (m^2^) as the sea level rises 0.3 m and 0.5 m, and a subsequent decrease under 1-m SLR.

### Deep Pool Habitat

This habitat type is the most at risk at all headlands, with 100% loss under 1-m SLR, at all headlands except Arrawarra Headland. The rate of habitat loss will differ between headlands, with Ocean View, Mullaway and Woolgoolga losing this habitat at 0.5-m SLR and Flat Top Point only at 1-m SLR.

### Upper Boulder Field Habitat

With the exception of Woolgoolga Headland, all headlands show a steady decline in the extent of upper boulder fields under 1-m SLR. At Woolgoolga Headland, habitat extent remains the same under 0.5-m SLR and then suffers a decrease of ∼50% under 1-m SLR. Flat Top Point will lose its entire upper boulder field habitat under 1-m SLR. While there is an overall decline at all headlands, this habitat type will suffer the second lowest overall loss (after lower platform habitat).

### Lower Boulder Field Habitat

Lower boulder field habitat shows an overall decline under 1-m SLR. At all headlands, nearly 50% of the habitat area is predicted to be lost under 0.3-m SLR. Flat Top Point shows the slowest decline in habitat area with around 30% remaining under 1-m SLR, whereas the other headlands will lose >85% of this habitat. Woolgoolga Headland will lose this entire habitat under 0.5-m SLR but will then recover 2% under 1-m SLR.

### Upper Platform Habitat

There will be a decrease in the area of upper platform habitat at all headlands over the next century (1-m SLR). Mullaway Headland will experience by far the greatest loss with 54% remaining under 0.3-m SLR, and 6% remaining under 1-m SLR. All other headlands show greater declines under 0.5-m SLR, with the exception of Ocean View Headland, which will retain 63% of upper platform habitat at 1-m SLR.

### Lower Platform Habitat

Lower platform habitat shows an irregular pattern of change over the next century. Arrawarra Headland will suffer a steady decline with <30% of habitat left under 1-m SLR. Flat Top Point will have a reduction of 50% under 0.5-m SLR, but very little subsequent change up to 1-m SLR. Mullaway Headland and Woolgoolga Headland will have an initial increase in habitat area at 0.5-m SLR, however, Mullaway Headland subsequently lose 18% (relative to the current area), whereas Woolgoolga Headland show a net 15% increase, under 1-m SLR. Ocean View will have the slowest rate of change with 76% of habitat area remaining under 1-m SLR.

## Discussion

It is clear from the results of this study that we can make few generalizations about the rate and extent of intertidal habitat loss resulting from SLR. While the extent of most habitats is ultimately reduced in the worst-case model (1-m SLR), the rate-of-change and final outcome are dependent on the habitat type and specific locality. In other words, the effects of SLR on intertidal rocky reefs will be variable at small spatial scales.

To date, most of the research conducted to evaluate the impact of SLR on intertidal ecosystems refers to mangroves [41], salt marshes [42]–[44] and sandy beaches [45]–[47]. Just a few studies have focused on the rocky shore ecosystem but have targeted exclusively large spatial scales, such as kilometers of coastal area [19]. By conducting studies at this level of spatial resolution (cm to m of habitat area), it has been possible to assess the relative vulnerability of different locations to habitat loss which may have important implications for conservation planning. Most headlands will show an increase in the ratio of lower shore to the upper shore, despite the overall reduction in intertidal area. Since lower shore habitats are known to be generally richer than the upper shore habitats [40] this may help to ameliorate the potential loss of biodiversity associated with the overall decline in intertidal area. Mean size and number of shallow pools will also show a variable, and headland-specific, change which would not have been detected by modelling at a larger scale (km of coastline) [19]. The conservation of fine-scale spatial heterogeneity is a critical factor in marine reserve planning [48] and, consequently, predictive modelling of SLR at broader scales will fail to effectively incorporate this feature into conservation targets.

An important extension of this is that, depending on the relative loss of critical habitat, headlands currently gazetted as sanctuary zones (no take) may not be effective locations to meet conservation targets in the future. If the objective is to protect representative habitats that are likely to persist, there is little point targeting headlands that will show high rates of habitat loss and ultimately support little habitat. It is instructive to apply this concept to the current conservation status of the five headlands. Both Flat Top Point and Arrawarra Headland are currently afforded the highest level of protection under the zoning plan for the Solitary Islands Marine Park (Special Purpose and Sanctuary, respectively) [49]. However, they will show some of the greatest reductions in the extent of shallow pools (upper and lower) and upper boulder field habitat, which are associated with high biodiversity [36]. The sea level projections from the present research have shown that these are two of the most vulnerable types of habitat and could nearly disappear under 1-m SLR, potentially exposing the resident fauna to considerable impact.

Eighty percent of the world's oceanic coastlines comprise of rock platforms backed by steep cliffs [19]; this description typifies headland formation for much of the Australian coastline [50] suggesting profound changes in the landscape with direct consequences for biodiversity and recreational use. The greatest challenge for coastal managers will be to evaluate how to deal with the lack of predictability for climate change factors [20], [21], since changes occur slowly and the effects also interact with other impacts already imposed on the environment such as pollution and anthropogenic disturbance [37], [51].

Present conservation planning is based on current sea levels. This leads to two questions: i) Will present sanctuary zones be effective for ongoing conservation in a changing environment?; ii) Does loss of habitat area equate to a commensurate loss of biodiversity for intertidal rocky reef ecosystems? Clearly, there is a need to determine if targets that foster prioritization of sites where habitat loss will be lower will actually translate into similar representation for the biota. This also needs to be considered in terms of other factors determining ecosystem functioning (e.g. various processes that can vary over a range of spatial scales) [52]. Environmental management in a changing world must, therefore, not only take into account new scenarios expected to arise over the next decades, but also be flexible enough to handle sudden change in predictions, which can hamper long-term biodiversity conservation [53].
